# Polysialylated-neural cell adhesion molecule (PSA-NCAM) in the human trigeminal ganglion and brainstem at prenatal and adult ages

**DOI:** 10.1186/1471-2202-9-108

**Published:** 2008-11-06

**Authors:** Marina Quartu, Maria Pina Serra, Marianna Boi, Viviana Ibba, Tiziana Melis, Marina Del Fiacco

**Affiliations:** 1 Department of Cytomorphology, University of Cagliari, Cittadella Universitaria di Monserrato, Monserrato (Cagliari), Italy

## Abstract

**Background:**

The polysialylated neuronal cell adhesion molecule (PSA-NCAM) is considered a marker of developing and migrating neurons and of synaptogenesis in the immature vertebrate nervous system. However, it persists in the mature normal brain in some regions which retain a capability for morphofunctional reorganization throughout life. With the aim of providing information relevant to the potential for dynamic changes of specific neuronal populations in man, this study analyses the immunohistochemical occurrence of PSA-NCAM in the human trigeminal ganglion (TG) and brainstem neuronal populations at prenatal and adult age.

**Results:**

Western blot analysis in human and rat hippocampus supports the specificity of the anti-PSA-NCAM antibody and the immunodetectability of the molecule in postmortem tissue. Immunohistochemical staining for PSA-NCAM occurs in TG and several brainstem regions during prenatal life and in adulthood. As a general rule, it appears as a surface staining suggestive of membrane labelling on neuronal perikarya and proximal processes, and as filamentous and dot-like elements in the neuropil. In the TG, PSA-NCAM is localized to neuronal perikarya, nerve fibres, pericellular networks, and satellite and Schwann cells; further, cytoplasmic perikaryal staining and positive pericellular fibre networks are detectable with higher frequency in adult than in newborn tissue. In the adult tissue, positive neurons are mostly small- and medium-sized, and amount to about 6% of the total ganglionic population. In the brainstem, PSA-NCAM is mainly distributed at the level of the medulla oblongata and pons and appears scarce in the mesencephalon. Immunoreactivity also occurs in discretely localized glial structures. At all ages examined, PSA-NCAM occurs in the spinal trigeminal nucleus, solitary nuclear complex, vestibular and cochlear nuclei, reticular formation nuclei, and most of the precerebellar nuclei. In specimens of different age, the distribution pattern remains fairly steady, whereas the density of immunoreactive structures and the staining intensity may change and are usually higher in newborn than in adult specimens.

**Conclusion:**

The results obtained show that, in man, the expression of PSA-NCAM in selective populations of central and peripheral neurons occurs not only during prenatal life, but also in adulthood. They support the concept of an involvement of this molecule in the structural and functional neural plasticity throughout life. In particular, the localization of PSA-NCAM in TG primary sensory neurons likely to be involved in the transmission of protopathic stimuli suggests the possible participation of this molecule in the processing of the relevant sensory neurotransmission.

## Background

The polysialylated form of the cell surface glycoprotein neural cell adhesion molecule (PSA-NCAM) is a dynamically regulated product of post-translational modification of NCAM [1,2]. Due to its large excluded volume, PSA can produce a sufficient physical hindrance between apposing membranes to attenuate intercellular adhesion [2,3]. The highest expression of PSA-NCAM occurs in the developing nervous system, where it is generally considered a promoter of neural plasticity, allowing migration of neural and nonneural precursors and facilitating axonal pathfinding and synaptogenesis [4,5]. In the normal adult brain of experimental animals NCAM generally displays low levels of polysialylation [6], with the exception of limited areas such as the hippocampus, the hypothalamus, the olfactory cortex and terminal regions of primary sensory afferents, which are believed to maintain a capability for morphological reorganization throughout life [7-10]. The PSA-NCAM levels and distribution have been shown to increase in learning and memory [11-13], chronic stress conditions [14-16] and several lesion models such as ischemia, epilepsy, brain trauma, and transected/crushed peripheral nerves [2,17-22]. Interestingly, the potential of PSA-NCAM expressing cells in promoting brain tissue repair [23-25] and a role for this molecule in neuroprotection [26] have been pointed out.

Data regarding the occurrence of the molecule in the normal human nervous system are still limited. Studies in the fetal brain focus on the localization of PSA-NCAM in discrete forebrain regions and on its role in developmental processes, such as neuronal migration and transitory axonal projections [27], and onset of myelination [28]. In the adult, evidence for the persistence of PSA-NCAM is restricted to the cerebral cortex [29-31] and peripheral nerve [32], where altered expression of the molecule has also been described in a number of neuropathological conditions [32-38]. In order to explore further the existence of neurons expressing the molecule in the human nervous system, thus providing potential for its dynamic changes and plasticity in response to environmental contests, we analysed whether PSA-NCAM occurs in the human primary sensory neurons, namely those of the trigeminal ganglion (TG), and in the morphofunctional heterogeneous populations of the brainstem. The study has been carried out by immunohistochemistry on normal tissue and conveys data on the occurrence of PSA-NCAM immunoreactivity in those regions at prenatal, neonatal and adult age. It may represent a basis for future analyses of pathological tissue specimens and, hopefully, for prospective applications in neuronal protection and repair. As the occurrence of PSA-NCAM and its persistence in the adult has been already shown in the human [29] and rat hippocampus [18,20], so as to test the antibody specificity and gain information on the immunodetectability of the molecule in autoptic versus non autoptic tissue, we also examined the detectability of PSA-NCAM in this brain region by western blot in both newborn and adult human and rat specimens.

## Results

### Western blot

**Figure 1:**
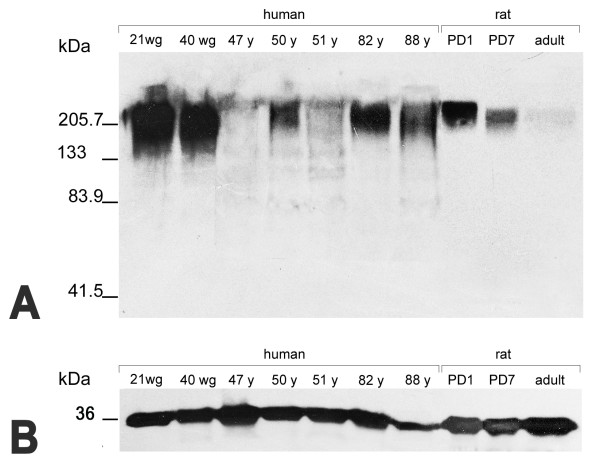
**Western blot analysis of PSA-NCAM (A) and GAPDH (B) in human and rat hippocampal tissue homogenates.** wg, weeks of gestation; y, years; PD, post-natal day.

In both the human and rat hippocampus tissue homogenates PSA-NCAM is immunochemically detectable as a single protein band whose level matches the expected molecular weight [39] (Figure 1). The band is present at all examined ages in both species. However its thickness and staining intensity are definitely more pronounced in the human pre- and full-term newborn versus adult tissue, as well as in the young versus adult rat tissue. A high degree of individual variability is also obvious among the adult human specimens.

### Immunohistochemistry

#### Trigeminal ganglion

**Figure 2:**
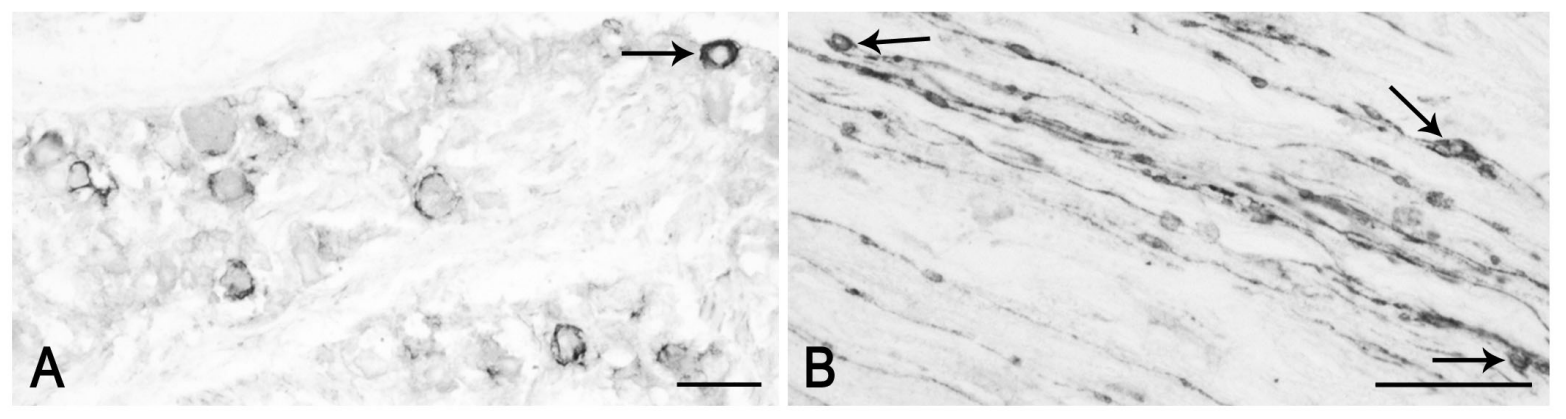
**PSA-NCAM in the human pre-term newborn trigeminal ganglion (case 3).** A: immunoreactivity of perikarya as peripheral or cytoplasmic (arrow) labelling. B: immunoreactive nerve fibres; arrows point to immunostained Schwann cells. Scale bars: A, B = 50 μm.

**Figure 3:**
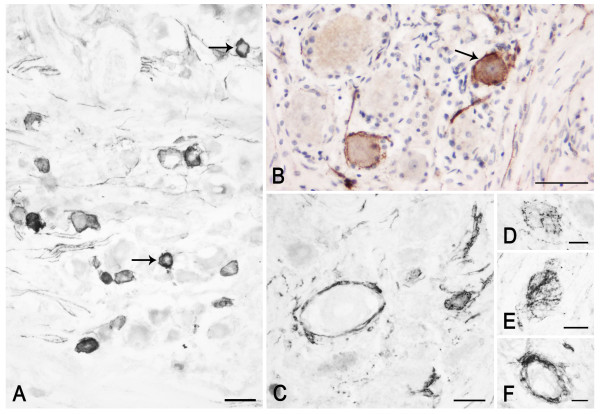
**PSA-NCAM in the human adult trigeminal ganglion (A, B: case 5, C-F: case 10).** A-C: immunostained perikarya and nerve fibres; in B, a tissue section immunostained for PSA-NCAM and co-stained with modified Mayer's hematoxylin is shown. Arrows in A and B point to PSA-NCAM immunostained neurons whose nucleus is clearly detectable. C, F: the immunolabelling appears localized to the satellite cells surrounding non immunoreactive neurons. D, E: pericellular varicose arborisations around non immunoreactive neurons. Scale bars: A-C = 50 μm; D = 25 μm; E, F = 20 μm.

PSA-NCAM is localized to neuronal perikarya, nerve fibres in bundles and in between neurons, and satellite and Schwann cells; further, intense cytoplasmic staining and networks of positive pericellular fibres occur in adult tissue and are detectable only occasionally in the newborn one (Figures 2, 3).

At fetal age, a number of neuronal cell bodies appear surrounded by immunoreactive material, which may be interpreted as membrane labelling, though aspects reminiscent of labelled satellite cells can be found; rare neurons show a cytoplasmic staining (Figure 2A).

**Figure 4:**
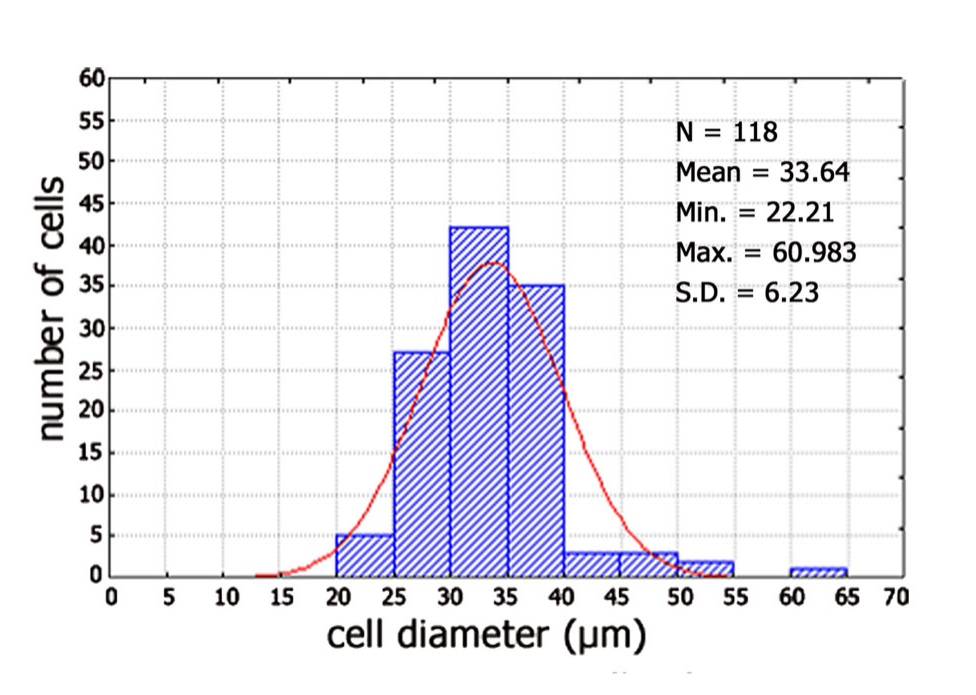
**Size frequency histogram of PSA-NCAM immunoreactive neurons in human trigeminal ganglion from an adult subject (case 5).** Cells present in 6 sections were measured. *x*-Axis values represent the mean cell diameters expressed in μm; *y*-axis reports values of relative percent frequency. Curve superimposed on the histogram represent the theoretical normal distribution.

In adult tissue, several neurons show a peripheral immunoreactivity suggestive of membrane labelling (Figure 3A,B). However, a number of them seem surrounded by immunostained satellite cells (Figure 3C,F), which makes it difficult to unequivocally identify a membrane staining and to ascertain a neuronal labelling. On the other hand, a number of perikarya also show a cytoplasmic labelling, thus allowing a morphometric analysis. This was done considering only the cell sections where both staining of the cytoplasmic compartment and the nucleus were clearly detectable, such as those indicated by arrows in Figure 3A,B. In the TG specimen from an adult subject (case 6), they amount to about 6.4 ± 0.075% of the total ganglionic population. Frequency histogram of those neurons is shown in Figure 4. About 70% of the sized neurons have a mean cell diameter ranging from 22 μm to 34 μm, thus falling in the class of small- and medium-sized cells, whereas the remaining of them fall in the large size range. Varicose fibres may be found either isolated or as bundles of variable density (Figure 3A–C). A few non immunolabelled neuronal cell bodies appear surrounded by immunoreactive varicose nerve fibres (Figure 3D,E).

#### Brainstem

**Figure 5:**
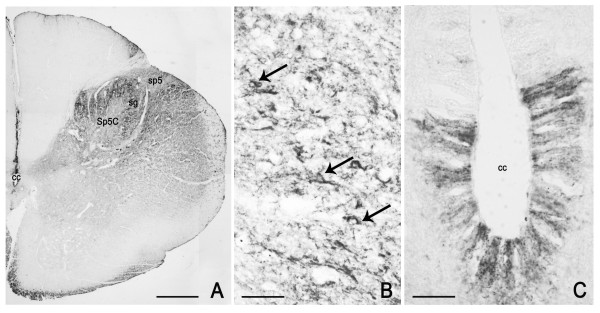
**PSA-NCAM in the human pre-term newborn medulla oblongata, caudal level (case 3).** A: panoramic view of the right half of a section at the boundary with the spinal cord. B: higher magnification of the substantia gelatinosa (sg) of the spinal trigeminal nucleus, caudal part (Sp5C); arrows point to labelled neuronal perikarya. C: ependymal lining of the central canal. cc; central canal; sp5, spinal trigeminal tract. Scale bars: A = 500 μm; B = 50 μm; C = 20 μm.

**Figure 6:**
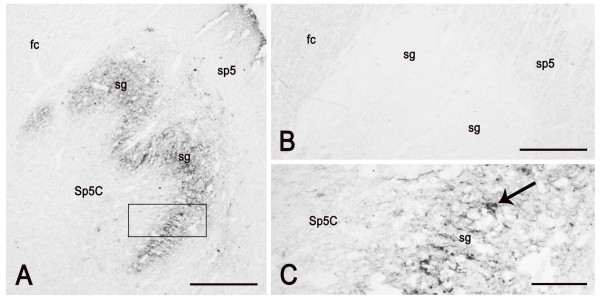
**PSA-NCAM in the human adult medulla oblongata, caudal level (case 10).** A: panoramic view of the right side spinal trigeminal nucleus, caudal part (Sp5C), at the boundary with the spinal cord. B: control immunostaining on a section semiconsecutive to that in A. C: higher magnification of the area framed in A; arrow points to a labelled neuron in the spinal nucleus substantia gelatinosa (sg). sp5, spinal trigeminal tract; fc, fasciculus cuneatus. Scale bars: A, B = 500 μm; D = 100 μm.

PSA-NCAM is mainly distributed at the level of the medulla oblongata and pons and appears scarce in the mesencephalon. The distribution pattern is uneven and remains fairly steady in specimens of different age, though changes in the density of immunoreactive structures and in the staining intensity may occur in newborn compared to adult specimens, as in the spinal trigeminal nucleus, caudal part (see Figures 5, 6). The vast majority of PSA-NCAM-immunolabelled structures appear as neuronal cell bodies and processes showing mainly a peripheral immunoreactivity suggestive of membrane labelling. Immunoreactivity also occurs in discretely localized glial structures, such as the dorsal median septum and the ependymal lining (Figure 5A,C). Moreover, in the latter, immunostained varicose thread-like elements may be seen running across the ependyma in a basoapical direction (Figure 5C).

**Figure 7:**
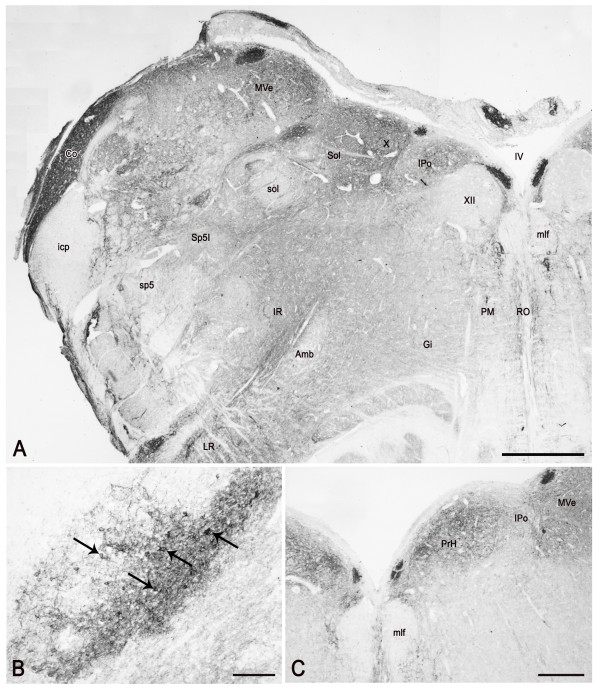
**PSA-NCAM in the human pre-term newborn medulla oblongata, rostral level (case 3).** A: panoramic view of the left side dorsal quadrant at the level of the rostral hypoglossal nucleus cell column. B: dorsal cochlear nucleus; arrows point to labelled neurons. C: low-power view of the dorsomedial field of the medulla oblongata at the level of the nucleus prepositus hypoglossi (PrH). Co, dorsal cochlear nucleus; Gi, gigantocellular nucleus; icp, inferior cerebellar peduncle; IPo, interpositus hypoglossi nucleus; IR, intermediate reticular nucleus; LR, lateral reticular nucleus; mlf, medial longitudinal fasciculus; mlf, medial longitudinal fasciculus; MVe, medial vestibular nucleus; PM, paramedian reticular nucleus; PrH, prepositus hypoglossi nucleus; RO, raphe obscurus nucleus; Sol, solitary nucleus; sol, solitary tract; sp5, spinal trigeminal tract; Sp5I, spinal trigeminal nucleus, interpolar part; IV, fourth ventricle; X, dorsal motor nucleus of the vagus nerve; XII, hypoglossal nucleus. Scale bars: A, C = 500 μm; B = 100 μm.

The trigeminal sensory nuclear complex shows an uneven distribution of immunoreactive elements. At all ages examined, PSA-NCAM is restricted to the spinal trigeminal nucleus (Figures 5, 6, 7A), where it is represented by neuronal cell bodies and processes, and is virtually absent in the principal and mesencephalic nuclei. At fetal age, the immunoreactivity labels fibres in the spinal tract of the trigeminal nerve (Figure 5A) and in the spinal nucleus pars caudalis, where a rich plexus and neuronal cell bodies occur in the substantia gelatinosa (Figure 5A,B), and in the pars interpolaris (Figure 7A). In adult tissue, the immunostaining persists in the substantia gelatinosa whereas the spinal tract and the magnocellular subnucleus harbor rare elements (Figure 6A,C).

**Figure 8:**
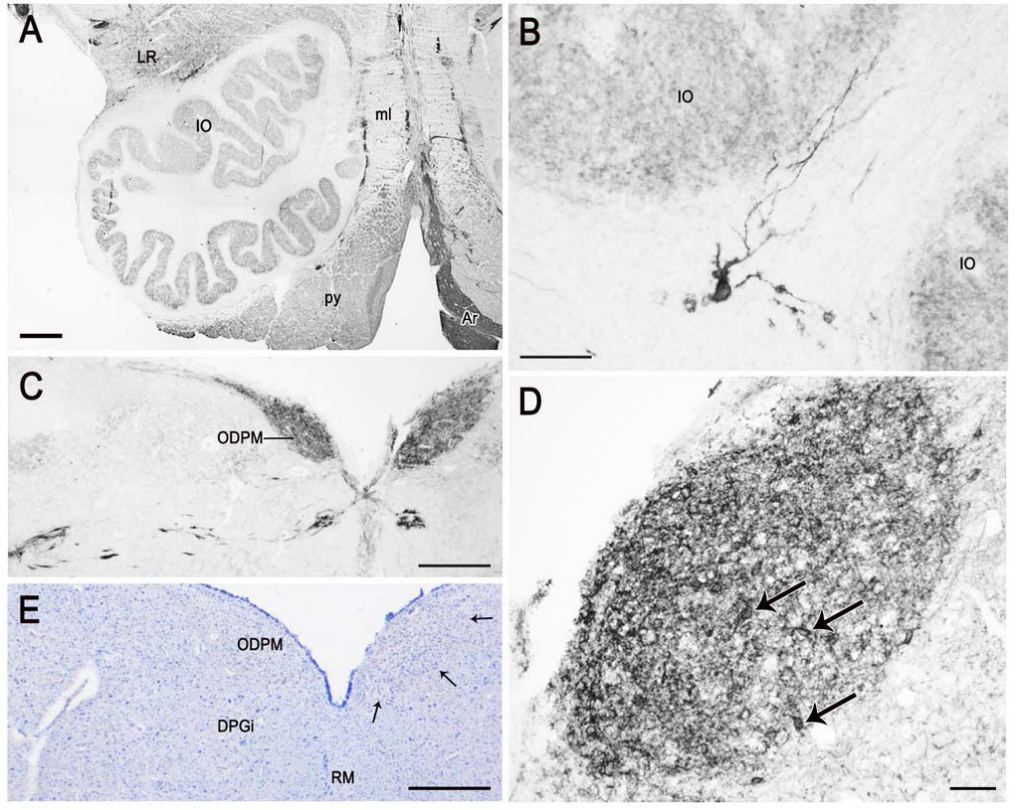
**PSA-NCAM in the human pre-term newborn rostral medulla oblongata (A-B) and caudal pons (C-E) (case 3). **A: left side ventral quadrant showing immunolabelling in the lateral reticular nucleus (LR), inferior olive (IO), pyramidal tract (py), and arcuate nucleus (Ar). B: a positive neuron in the white matter around the inferior olive, which extends long labelled processes towards the nucleus. C: low-power view of the dorsomedial field of the medulla oblongata including the dorsal paramedian nucleus, oral part (ODPM). D: higher magnification of the right side dorsal paramedian nucleus, oral part; arrows point to positive neuronal perikarya. E: modified Mayer's hematoxylin counterstaining of a tissue section semiconsecutive to that shown in C and adjacent to that shown in D; small arrows outline the cytoarchitectonic boundaries of the immunoreactive area shown in D. DPGi, dorsal paragigantocellular nucleus; ml, medial lemniscus; RM, raphe magnus nucleus. Scale bars: A, C, E = 500 μm; B = 20 μm; D = 50 μm.

**Figure 9:**
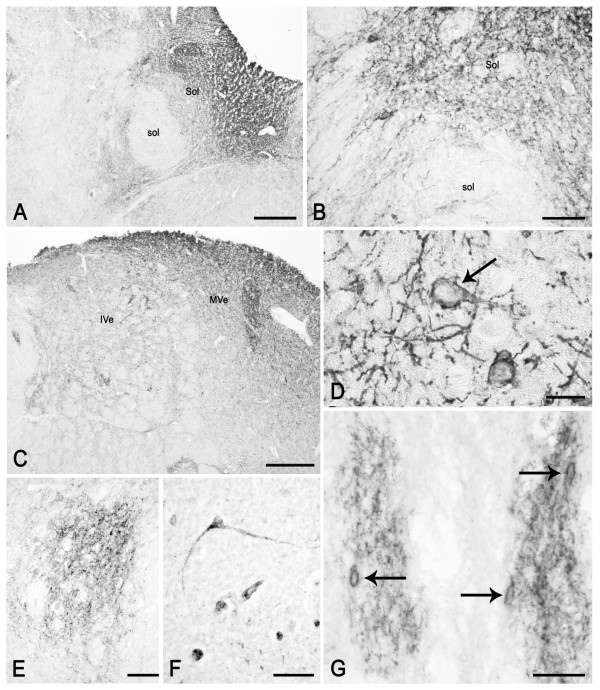
**PSA-NCAM in the human adult medulla oblongata (case 5). **A, B: left side solitary nucleus (Sol) and tract (sol). C: left side inferior (IVe) and medial vestibular (MVe) nuclei. D: a neuronal perikaryon with membrane labelling (arrow) and fibres in the neuropil of the medial vestibular nucleus. E: hypoglossal nucleus. F: dorsal motor nucleus of the vagus nerve. G: perikarya with membrane labelling (arrows) and fibre networks in the raphe obscurus nucleus. Scale bars: A, C, E = 500 μm; B = 100 μm; D = 20 μm; F, G = 50 μm.

At caudal level of the medulla oblongata, besides the spinal trigeminal nucleus which harbors the bulk of immunoreactivity, the remaining grey substance shows a very light to moderate immunostaining in the territory of the supraspinal, central reticular and lateral reticular nuclei. Close to the ventral surface of the anterior funiculus, a band of intensely immunoreactive nerve fibres can be observed (Figure 5A). More rostrally, at the level of the pyramidal decussation, the gracile, external cuneate and commissural nuclei show a moderate immunolabelling in form of loose plexuses of filamentous and dot-like elements. A moderate diffuse immunostaining is detectable in the area postrema. Above the obex, at the level of the inferior olive, the tegmentum shows several areas of intense to moderate immunoreactivity encompassing both cranial nerve and reticular formation nuclei (Figures 7, 8, 9). At all ages examined, neuronal perikarya and plexuses of labelled nerve fibres can be observed in the solitary nuclear complex (Figures 7A, 9A,B), the vestibular (Figures 7A, 9C,D) and cochlear nuclei (Figure 7A,B), and the dorsal motor nucleus of the vagus nerve (Figure 7A, 9F). While its immunoreactivity is scarce in the newborn (Figure 7A), in the adult, the hypoglossal nucleus shows a moderate immunostaining in form of nerve fibre plexuses surrounding non immunoreactive neuronal cell bodies (Figure 9E). Positive fibre networks and peripherally labelled neurons are located in the intercalatus and prepositus hypoglossi (Figure 7A,C), the paramedian reticular (Figure 7A), the raphe obscurus (Figure 7A, 9G), the gigantocellular, the central reticular, the intermediate reticular and the lateral reticular nuclei (Figure 7A). The olivary nuclear complex shows a diffuse staining in the neuropil at all ages (Figure 8A) with some small cell bodies in adult specimens. At fetal age, the white matter surrounding the inferior olive harbors isolated large, intensely stained neurons which extend dendritic branches towards the nucleus (Figure 8B). The arcuate nucleus appears intensely labelled (Figure 8A). In newborn specimens, the pyramidal tract is moderately stained (Figure 8A). Nerve fibres, isolated or grouped in small bundlets, occur across the medial lemniscus (Figure 8A).

In the pons, bundles of strongly immunoreactive nerve fibres run in the eighth cranial nerve and reach the pontine territory of the vestibular and cochlear nuclei, which harbor immunoreactive cell bodies. In the newborn, at caudalmost levels, the oral part of the dorsal paramedian nucleus (Figure 8C) shows an intense immunoreactivity due to positive neuronal perikarya and processes (Figure 8D). The subependymal grey contains a light punctate labelling throughout. In the adult, a diffuse weak staining is also appreciable in the locus caeruleus neuropil in between the unstained pigmented neurons. The caudal pontine, gigantocellular, parvocellular, reticulotegmental, and oral pontine reticular nuclei contain sparse neuronal cell bodies with peripheral membrane labelling and rare punctate and filamentous elements. The pontine nuclei show an intense labelling due to peripherally stained neuronal perikarya and a meshwork of thin filamentous and punctate elements in between them. Labelled nerve fibres, isolated or in bundlets, occur across the medial longitudinal fasciculus, the medial lemniscus and the lateral lemniscus.

In the quadrigeminal plate, sparse neuronal perikarya occur in the external and pericentral nuclei of the inferior colliculus, the intercollicular region and the superior colliculus. A diffuse immunostaining, occasionally localized to neuronal perikarya, occurs in the periacqueductal grey and in the territory of the median and dorsal raphe nuclei. Only in adult tissue a diffuse immunostaining can be observed in the substantia nigra. The cerebral peduncle harbors transversely sectioned labelled fibres. Close to the ventral surface of the caudal mesencephalon, positive nerve fibres form a small area of dense immunoreactivity located along the lateral edge of the interpeduncular fossa.

## Discussion

The results obtained indicate that in the human nervous system a subpopulation of the TG primary sensory neurons and several regions of the brainstem express PSA-NCAM throughout life. Centrally, the sialylated protein appears associated with most of the sensory nuclei, in agreement with findings on laboratory animals and the concept that, in these regions, the expression of growth-related proteins may subserve structural reorganization and synaptic plasticity in response to afferent activity [18,40] throughout life [20]. As for the spinal trigeminal sensory system, occurrence of the growth-associated protein-43 (GAP-43) has been reported in both the sensory ganglion and the central nuclei [41]. The localization of PSA-NCAM to the caudal part of the trigeminal spinal sensory nucleus and its changes with age are consistent with data on the rat dorsal horn spinal cord showing that PSA-NCAM is more widely expressed during embryonic and early postnatal than in adult life, when it is confined to the superficial laminae [42,43]. Such a discrete central localization of the molecule together with the morphometric characteristics of the immunoreactive trigeminal primary sensory neurons point to an involvement of PSA-NCAM in the functional roles of the elements drawn in the neurotransmission and processing of protophatic sensory stimuli. It is interesting that a PSA-dependent reversible loss of C terminals occurs in the spinal lamina II in a model of chronic neuropathic pain [10]. In this context, it has been proposed that, under injury or stress conditions, the presence of PSA allows for a local and reversible break in the afferent pathway in response to excessive stimulation and thereby could serve to protect central circuitry from chronic sensory overload [10].

PSA-NCAM has been shown in the chicken acoustic ganglion cells [8], where a role for the molecule in the ganglion neuron plasticity and in the processing of auditory information has been suggested. Afferents to the mouse ventral cochlear nucleus express high levels of PSA [10] and, similarly to the structural plasticity of nociceptive C terminals in chronic neuropathic pain [10], it has been reported that noxious acoustic insults are associated with a reversible atrophy of nerve terminals in the ventral cochlear nucleus [44-46].

Our observations also indicate that at all ages examined the dorsal vagal complex, namely the nucleus of the solitary tract, the dorsal motor nucleus of the vagus nerve, and the area postrema, contains PSA-NCAM-labelled neurons, nerve fibres and terminals. A convergent set of experimental data shows that in these areas, though visceral sensorimotor circuits are morphologically established in newborn rats [47,48], intense dynamic changes of neuronal properties still occur after birth [40,49-54]. In the rat solitary tract nucleus, the overall PSA-NCAM expression decreases during the first two postnatal weeks and persists only at synapses in the adult [40]. Furthermore, PSA-NCAM expression has been shown to be dynamically controlled by the electrical stimulation of the vagal afferents [40] and, conversely, a repetitive stimulation of afferent fibres leads to phasic and long-term plasticity in adult animals [55].

It has been shown that the rat raphe serotonin neurons do not express PSA-NCAM [56]. Thus, it may be considered that the staining on the raphe neuronal perikarya observed in our specimens either belongs to those neurons, marking a difference with the rat species, or reflects the occurrence of positive elements impinging on the raphe neuronal somata.

PSA-NCAM occurs in most of the precerebellar nuclei, which act as a gate for the input to the cerebellum. Its expression appears particularly robust in nuclei which play a critical role in eye movement control, such as the intercalatus and prepositus hypoglossi nuclei and the paramedian reticular nucleus [57,58].

PSA-NCAM-related neuroplasticity may also involve glial cells. Several lines of evidence indicate that PSA-NCAM plays a permissive role for the structural remodelling of neuronal and glial cells, particularly in the neuroendocrine system, where PSA-NCAM appears to control the retraction of the glial processes in the hypothalamo-neurohypophysial system [18,59]. Moreover, changes in PSA-NCAM in the avian ciliary ganglion after axonal injury also involve perineuronal satellite cells [60]. In agreement with the steric hindrance caused by the molecule [2,3], the occurrence of PSA-NCAM in the TG may indicate sites of detachment between the neuronal surface and its glial ensheatment. Dynamic changes, such as remodelling of the perikaryal surface that may be operated via other growth-related proteins such as GAP-43 [61], may be facilitated by this effect. As for the finding of PSA-NCAM-positive ependymal cells in the central canal lining, it is possible that we are observing a subpopulation of glial cells which, as recently suggested in the human brain [62], during gestation and up to early infancy remains undifferentiated as potential neural progenitor cells.

## Conclusion

The expression of PSA-NCAM in selective populations of central and peripheral neurons indicate that the molecule occurs both during prenatal life, suggesting a role in the development and/or maturation of neuronal circuitry, and in adulthood, when it may be indicative of the capacity to structural and functional neuronal plasticity and is possibly involved in the processing of sensory information throughout life. The neurochemical characterization and connectivity of the PSA-NCAM-positive neurons is an important issue and deserves further investigation. The localization of PSA-NCAM in primary sensory neurons likely to be involved in the transmission of protopathic stimuli bears a specific significance as it suggests the possible participation of this molecule in the processing of the relevant sensory neurotransmission.

## Methods

Specimens of trigeminal ganglion, brainstem segments, and hippocampus were obtained at autopsy from subjects of different ages, with no history of neuropathology (Table 1). The sampling and handling of human specimens conformed to the local Ethics Committee of the National Health System in compliance with the principles enunciated in the Declaration of Helsinki. The hippocampus of male Sprague-Dawley rats sacrificed by decapitation at post-natal day (PD) 1 (2 rats), PD7 (2 rats) and adult age (2 rats) was also sampled for western blot analysis and stored at -80°C until required.

**Table 1:** List of specimens

**Case**	**Age**	**Sex**	**Cause of death**	**Post-mortem delay**	**Method**	**Specimen**
1	21 w.g.	M	Cardio-respiratory failure	35 h	WB	Hippocampus
2	28 w.g	F	Cardio-respiratory failure	37 h	IHC	Brainstem, TG
3	36 w.g.	F	Cardio-respiratory failure	26 h	IHC	Brainstem, TG
4	40 w.g.	M	Cardio-respiratory failure	37 h	WB	Hippocampus
5	42 y	M	Cardio-respiratory failure	37 h	IHC	Brainstem, TG
6	47 y	F	Pneumonitis	50 h	WB, IHC	Hippocampus, brainstem, TG
7	50 y	F	Embolysm of pulmonary artery	26 h	WB	Hippocampus
8	51 y	F	Myocardial infarction	54 h	WB, IHC	Hippocampus, brainstem
9	82 y	F	Myocardial infarction	28 h	WB	Hippocampus
10	88 y	M	Embolysm of pulmonary artery	47 h	WB, IHC	Hippocampus, brainstem, TG

F, female; h, hours; IHC, immunohistochemistry; M, male; TG, trigeminal ganglion; w.g., weeks of gestation; WB, western blot.

### Western blot

Tissue blocks of hippocampus from a pre-term newborn (case 1), a full-term newborn (case 5) and five adult subjects (cases 6, 7, 8, 11, 12) were collected and stored at -80°C until required. Human and rat tissue homogenates were prepared in 50 mM Tris HCl, pH 7,5, centrifuged at 10,000 × g for 20 min, resuspended in 3 ml of Tris HCl, pH 7,5. Protein concentrations were determined using the Lowry method of protein assay [63] with bovine serum albumin as standard. Proteins for each tissue homogenate (30 μg) diluted 1:1 in loading buffer were heated to 95°C for 3 min and separated by SDS-polyacrilamide gel electrophoresis (SDS-PAGE) using 8.5% (w/v) polyacrilamide resolving gel. Internal molecular weight standards (Kaleidoscope Prestained Standards, Bio-Rad, Hercules, CA, USA) were run in parallel. Two gels at a time were run for Coomassie staining and immunoblotting, respectively. Proteins for immunoblotting were electrophoretically transferred on a polyvinylidene fluoride membrane (Biorad) using the Mini Trans Blot Cell (Biorad). Blots were blocked by immersion in 20 mM Tris base and 137 mM sodium chloride (TBS) containing 5% milk powder and 0.1% Tween 20 (TBS-T), for 60 min at room temperature and incubated overnight at 4°C with the primary antibody. A mouse monoclonal antibody against PSA-NCAM (Chemicon, USA), diluted 1:5,000 in TBS containing 5% milk powder and 0.4% Micro-O-Protect (MOP) (Boehringer Mannheim), was used as primary antiserum. After TBS-T rinse, blots were incubated for 60 min, at room temperature, with a peroxidase-conjugated anti-mouse serum (Chemicon), diluted 1:10,000 in TBS/T. Loading controls were obtained by stripping and immunostaining the membranes as above, using a monoclonal mouse antibody against glyceraldehyde 3-phosphate dehydrogenase (GAPDH) (Chemicon), diluted 1:600, as primary antiserum, and a peroxidase-conjugated goat anti-mouse serum (Chemicon), diluted 1:10,000, as secondary antiserum. In order to control for non specific staining, blots were stripped and incubated with the relevant secondary antiserum. After TBS-T rinse, protein bands were visualized on a film (Kodak X-Omat LS, Kodak, Rochester, NY) using the ECL method (Amersham Corp.).

### Immunohistochemistry

Human TG and brainstem specimens were fixed by immersion in 4% freshly prepared phosphate-buffered formaldehyde, pH 7.3, for 4–6 hours at 4°C, and rinsed overnight in 0.1 M phosphate buffer (PB), pH 7.3, containing 10% or 30% sucrose for adult and newborn specimens, respectively. Cryostat sections 14 μm thick were collected on chrome alum-gelatin coated slides and processed by the avidin-biotin-peroxidase complex (ABC) immunohistochemical technique. The same anti-PSA-NCAM antibody used for the Western blot analysis, diluted 1:400, was applied. Biotin-conjugated goat anti-mouse serum (Vector), diluted 1:300, was used as secondary antiserum. The reaction product was revealed with ABC (BioSpa Div.), diluted 1:250, followed by incubation with a solution of 0.1 M PB, pH 7.3, containing 0.05% 3-3'-diaminobenzidine (Sigma), 0.04% nickel ammonium sulphate, and 0.01% hydrogen peroxide. Incubations with primary antiserum were carried out overnight at 4°C. Incubations with secondary antiserum and ABC lasted 70 and 30 minutes, respectively, and were performed at room temperature. All antisera and the ABC were diluted in phosphate buffered saline containing 0.2% Triton X-100 (PBS/T). Control immunostainings were obtained either by omitting the primary antibody or by substituting it with normal goat serum. Alternate sections were stained with modified Mayer's hematoxylin and examined with reference to brainstem maps in Nieuwenhuys et al. [64] and Paxinos et al. [65] for the territorial identification. Slides were dehydrated, cover-slipped, and observed with an Olympus BX61 microscope. Digital images were captured with a ColorViewII Olympus telecamera.

### Morphometry

Morphometric analysis was performed on TG neuronal cell profiles of digital images captured with a 20× objective magnification. Tissue sections distant from each other at least 84 μm and only cells in them that obviously showed the nuclear profile were considered. Neuronal mean diameters were automatically evaluated by ImageProPlus software. Statistical parameters (mean, median, S.D.) and histograms were obtained by Statistica 6 software. The percentage of positive perikarya was calculated by the ratio of the total number of labelled cells found in three to six sections to the total number of cells found in the same sections after a modified Mayer's hematoxylin counterstaining.

## Authors' contributions

MDF conceived and coordinated the study, and revised the manuscript. MQ assisted in coordinating the study, performed the statistical analysis and wrote the manuscript. MPS performed the western blot analysis and assisted in immunohistochemistry. MB performed immunohistochemistry and assisted in statistical analysis. VI and TM assisted in immunohistochemistry. All authors read and approved the final manuscript.
